# Iron metabolism disorders of patients with chronic paracoccidioidomycosis

**DOI:** 10.1371/journal.pone.0282218

**Published:** 2023-06-22

**Authors:** Eliana da Costa Alvarenga de Brito, Igor Valadares Siqueira, James Venturini, Vinícius Lopes Teodoro Félix, Alana Oswaldina Gavioli Meira dos Santos, Rinaldo Poncio Mendes, Simone Schneider Weber, Anamaria Mello Miranda Paniago

**Affiliations:** 1 Graduate Program in Infectious and Parasitic Diseases, Faculty of Medicine, Federal University of Mato Grosso do Sul, Campo Grande, Mato Grosso do Sul, Brazil; 2 Scientific Initiation CNPq, Faculty of Medicine, Federal University of Mato Grosso do Sul, Campo Grande, Mato Grosso do Sul, Brazil; 3 Graduate Program in Health and Development of the Central West Region, Federal University of Mato Grosso do Sul, Campo Grande, Mato Grosso do Sul, Brazil; 4 Department of Tropical Diseases, Botucatu Medical School, Universidade Estadual Paulista (UNESP), Botucatu, São Paulo, Brazil; 5 Faculty of Pharmaceutical Sciences, Food and Nutrition, Federal University of Mato Grosso do Sul, Campo Grande, Mato Grosso do Sul, Brazil; University of the Witwatersrand, SOUTH AFRICA

## Abstract

Paracoccidioidomycosis (PCM) is caused by *Paracoccidioides* spp.; during infection, some host mechanisms limit the availability of iron, thereby reducing its reproduction. However, *Paracoccidioides* spp. can evade the immune defense and, even under limited iron conditions, use this mineral for growth and dissemination. This study evaluated the iron metabolism of 39 patients who were diagnosed with chronic PCM from 2013 to 2021. The forms of iron before treatment and at the time of clinical cure were evaluated based on the following: serum ferritin levels (storage iron); total iron-binding capacity (TIBC) and transferrin saturation (TSAT) level (transport iron); red blood cell (RBC), hemoglobin (Hb), hematocrit (HCT), and soluble transferrin receptor (sTfR) levels; and sTfR/log ferritin ratio (functional iron). The mean age of the patients was 54.5 years (±6.7 years). Most patients were men (97.4%), rural workers (92.1%), and smokers (84.6%); furthermore, most had moderate disease severity (66.7%). After achieving clinical cure, we observed that serum ferritin levels decreased, and parameters of functional iron increased. The extent of alteration in these parameters were more pronounced in severe cases than in to mild or moderate cases. Furthermore, moderate correlations were observed between C-reactive protein and the Hb (r = -0.500; p = 0.002), RBC (r = -0.461; p = 0.005), HCT (r = -0.514; p = 0.001), and iron levels (r = -0.491; p = 0.002). However, it is possible to infer that PCM interferes with functional and storage iron because improvements in these parameters after treatment as well as associations with disease severity were observed. PCM can lead to anemia of inflammation, which can be differentiated from iron deficiency anemia by a careful investigation of the iron form parameters.

## Introduction

Paracoccidioidomycosis (PCM), is a systemic mycosis endemic to Latin America that, is caused by *Paracoccidioides* spp., which is a dimorphic fungus. Infection occurs through the inhalation of infective conidia that are present in the soil; these conidia settle in the lungs and can spread to the lymphatic and hematogenous pathways and other organs. PCM is considered the eighth most common cause of death attributable to chronic infectious diseases in Brazil [1]. Chronic or adult PCM, which is observed in those older than age 30 years, is the most frequent form of this disease, and it predominantly affects the lungs and mucous membranes of the upper digestive tract and airways [2].

Similar to other fungal diseases, certain factors are determinants of PCM, such as the inoculum size, pathogenicity, fungus virulence, defense system integrity, and genetic factors [3]. The host organism uses strategies or barriers that prevent fungal diseases, such as inhibiting vital fungal functions by blocking iron uptake; this is because iron is an essential mineral for the proliferation of many pathogens, including *Paracoccidioides* spp. [4].

Iron is an essential component of hemoglobin (Hb), myoglobin, and several enzymes, and it has a fundamental role in oxygen transport and electron transfer. Additionally, it acts as a cofactor in many enzymatic processes, including DNA synthesis [5–7].

Maintaining control of iron homeostasis is essential in host-pathogen interactions because both compete for this micronutrient. During infection, some host immune mechanisms limit the availability of iron to invading microorganisms, thereby reducing their proliferation [8]. However, many microorganisms, such as *Paracoccidioides* spp., can evade the immune defense; even under limited iron conditions, they can use this mineral for growth and dissemination [9]. Therefore, alterations in hematological parameters and iron metabolism can be observed during PCM infection [10].

In the human body, iron is distributed in different interconnected forms to enable homeostasis. Storage iron refers to iron stored for future use in the body. Ferritin, a major iron-storage protein, forms a spherical structure and stores iron in its stable Fe3+ form. It acts as a buffer by, regulating iron release to prevent iron overload or deficiency. Transport iron refers to iron bound to transferrin, a protein that carries iron in the bloodstream to cells that need it for physiological processes. Transferrin binds to iron with high affinity, thus preventing the formation of harmful reactive oxygen species Circulating transferrin can be evaluated by total iron binding transferrin (TIBC). The functional iron compartment includes iron involved in hemoglobin synthesis and erythropoiesis, which is the production of red blood cells. Iron is essential for proper hemoglobin function and erythropoiesis processes [11].

Storage iron, transport iron, and functional iron forms can be evaluated separately using laboratory tests, and many of these tests are available and accessible during routine laboratory evaluations. These tests can assess any disturbances in iron metabolism and the affected iron form [11]. Generally, when there is an iron deficit, these iron forms are sequentially affected. First, there is a decrease in the storage iron levels, followed by a deficiency in the transport iron level and a reduction in the functional iron level [12, 13].

The host-parasite interaction and consequent inflammatory response lead to changes in iron metabolism dynamics. These changes can be observed in some patients with PCM [14], which is a chronic systemic inflammatory disease requiring long-term treatment.

Because of the gap in the scientific literature regarding some iron metabolism parameters of chronic PCM, this study aimed to update this topic and enhance PCM management.

## Materials and methods

### Ethical aspects

This study was approved by the Human Research Ethics Committee of the Federal University of Mato Grosso do Sul (CAAE number 62726016.2.0000.0021). All participants provided written informed consent.

### Location, period, and design of the study

This study was performed from 2013 to 2021 at the outpatient clinic for systemic mycoses of the Infectious and Parasitic Diseases Unit of the University Hospital Maria Aparecida Pedrossian at the Federal University of Mato Grosso do Sul, which is a reference center for infectious diseases, in Campo Grande, Mato Grosso do Sul, Brazil. This analytical study involving prospective data evaluated iron metabolism parameters of patients with chronic PCM before treatment and at the time of clinical cure.

### Patients

#### Inclusion and exclusion criteria

Male and female individuals who were diagnosed with chronic PCM from September 2013 to February 2021 were included. Patients with chronic PCM who died and/or did not undergo laboratory tests at admission, such as serum iron and blood count (hemogram [HMG]) tests, were excluded, as were patients diagnosed with human immunodeficiency virus, tuberculosis, and/or neoplasia.

#### Case definition

Confirmed cases of PCM were those presenting with suggestive clinical manifestations, typical yeast forms of *Paracoccidioides* spp. visualized by mycological, culture, or direct histopathological tests, and/or serum-specific antibodies detected using the double agar gel immunodiffusion test.

### Demographic and clinical variables

Demographic, clinical, and laboratory data were retrieved from a prospective database. They comprised complete routine medical data obtained at the time of diagnosis and during follow-up of the disease. Demographic data, such as sex, age, occupational history, smoking, and clinical disease severity, were collected before treatment.

Chronic PCM was subdivided into mild, moderate, and severe cases. Mild cases included body mass index (BMI) reduction <5% of the usual BMI and the involvement of one or a few organs without functional changes. Severe cases comprised three or more of the following criteria: BMI reduction ≥10% of the usual BMI; severe pulmonary impairment; involvement of other organs, such as the adrenal glands, central nervous system, and bones; enlargement of multiple lymph node chains (superficial or deep chains with pseudotumors [>2.0 cm in diameter] with or without suppuration); and high-titer antibodies. Moderate cases were defined as intermediate cases that were worse than mild cases but not as serious as severe cases [2].

### Laboratory analysis

Blood samples were obtained during the routine medical examination at the time of diagnosis and during follow-up. Laboratory data and complementary laboratory test data were obtained at two stages of routine outpatient follow-up: before treatment and at the time of clinical cure (disappearance of signs and symptoms) [2]. To investigate the iron metabolism of patients with chronic PCM, different forms of iron were evaluated.

### Storage iron

To assess the storage iron, serum ferritin levels were measured using an electrochemiluminescence immunoassay (Roche).

### Transport iron

To assess the transport iron the total iron-binding capacity (TIBC) was measured using a colorimetric assay (Roche) and the transferrin saturation (TSAT) level. The TSAT level was calculated as [TSAT = serum iron/TIBC × (100)], and the result was expressed as a percentage (%).

### Functional iron

To evaluate functional iron, the blood count was evaluated using the HMG test to measure the Hb level, red blood cell (RBC) level, mean corpuscular volume, and mean Hb concentration (MHC) (XN 3000 series hematology equipment; Sysmex Corporation, Kobe, Japan). Serum iron was determined using a colorimetric assay (Roche). The soluble transferrin receptor (sTfR) level was determined using an enzyme-linked immunosorbent assay (human sTfR ELISA Kit; Elabscience). This test is not routinely performed; therefore, serum stored in the PCM biobank was used.

The inflammatory process was assessed according to the high-sensitivity serum C-reactive protein (CRP) level measured using immunoturbidimetry. All analyzed parameters and values considered normal by the manufacturer are listed in **Table 1**.

**Table 1:** Iron parameters and reference values.

Parameters	Reference values
**Storage iron**	
Serum ferritin*	Males: 30–400 ng/mL
	Females: 13–150 ng/mL
**Transport iron**	
Transferrin saturation*	30%-50%
Total iron-binding capacity*	250–410 μg/dL
**Functional iron**	
Total erythrocyte count (red blood cells)#	Males: 4.5–5.5 million/mm^3^
	Females: 3.8–4.8 million/mm^3^
Hemoglobin#	Males: 13.0–17 g/dL
	Females: 12.0–15 g/dL
Hematocrit#	Males: 40%-50%
	Females: 38%- 45%
Mean corpuscular volume#	80–101 fL
Mean hemoglobin concentration#	27–32 pg
Serum iron*	61–157 μg/dL
Soluble transferrin receptor*	16.79–172.63 ng/mL
**Inflammatory process**	
C-reactive protein*	0–5.0 mg/L

*Reference values provided by the manufacturer.

^#^Reference values provided by Kawthalkar, 2013 [15].

### Statistical analysis

The statistical data analysis was performed using Jamovi software (version 1.6) for Windows [16]. Data are presented as the mean ± standard deviation (SD) or median with the first quartile (Q1) and third quartile (Q3). Comparisons between values before treatment and at the time of clinical cure were performed using Student’s t test or the Wilcoxon signed rank test. The Pearson correlation coefficient I was used to determine the relationship between variables; values ≤0.29, values between 0.30 and 0.49, and values between 0.50 and 1.0 were considered weak, moderate, and strong correlations, respectively.

McNemar’s test was performed to assess the associations between qualitative variables and Kruskal-Wallis test was performed to compare quantitative variables in more than two independent groups The Shapiro-Wilk test was performed to determine whether the variables were normally distributed.

A proportion test and one-sample test were performed to analyze the categorical and numerical variables, respectively. For all tests, p ≤ 0.05 was considered significant.

## Results

**Fig 1:**
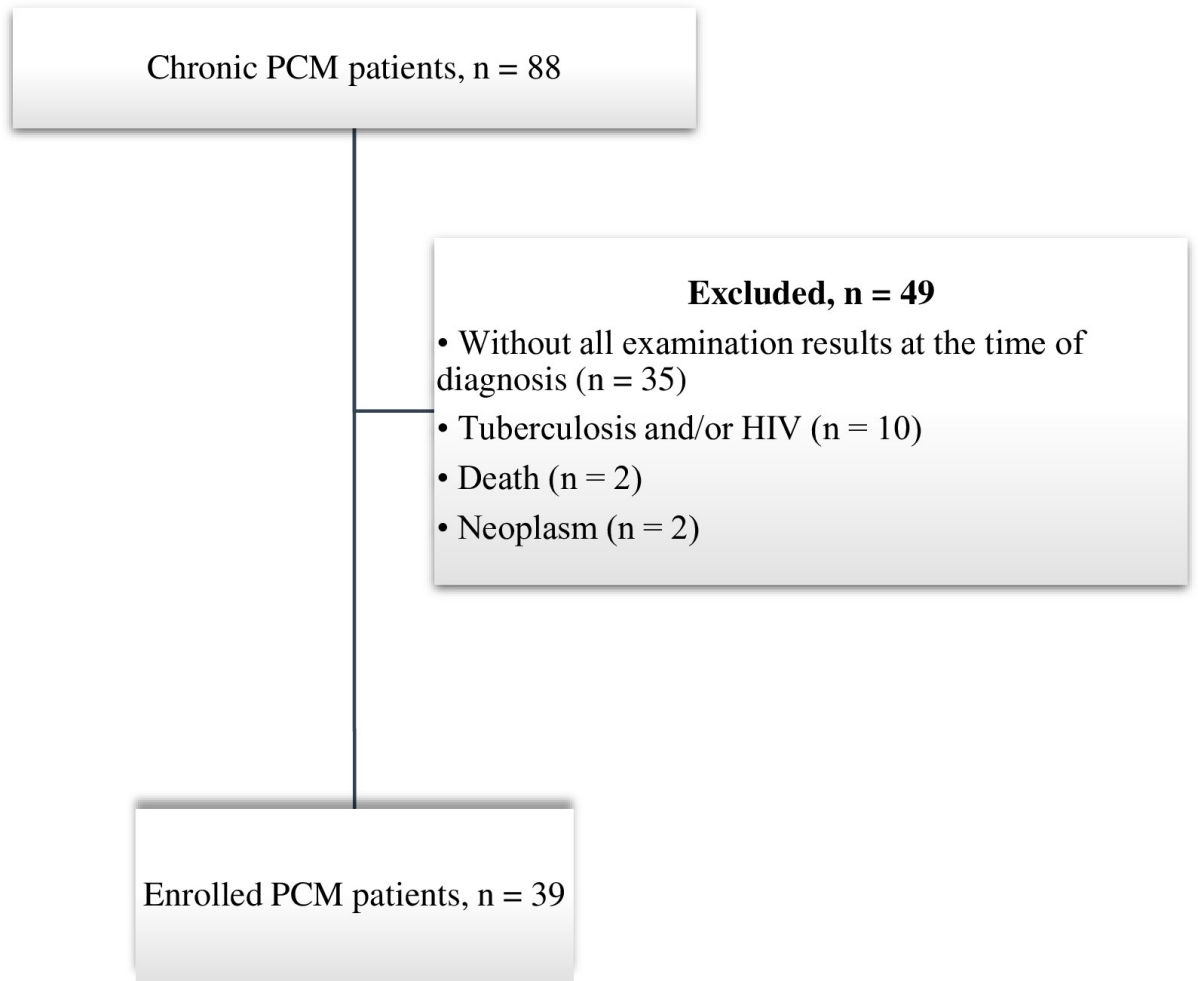
Flowchart of the selection of participants with chronic paracoccidioidomycosis (PCM) from 2013 to 2021. HIV, human immunodeficiency virus.

A total of 88 patients were diagnosed with chronic PCM during the study period. Of these 88 patients, 35 patients were excluded according to the criteria and 39 patients participated in the study (**Fig 1**). The mean age of these patients was 54.5 years (SD, 6.70 years); 38 (97.4%) were men and 35 (92.1%) were rural workers. Thirty-three (84.6%) individuals were smokers at the time of diagnosis. Regarding disease severity, 66.7% of the patients had moderate disease, 20.5% had severe disease, and 12.8% had mild disease (**Table 2**).

**Table 2:** Baseline demographic, clinical, and lifestyle data of 39 patients with chronic paracoccidioidomycosis.

Variables	n (%)	95% CI		p
**Sex**				<0.001
Male	38 (97.4)	86.5	99.9	
Female	1 (2.6)	0.06	13.5	
Age (in years)*	54.5 ± 6.70	52.3	56.6	<0.001†
**Rural workers (n = 38)**				<0.001
Previous/current	35 (92.1)	78.6	98.3	
Never	3 (7.9)	1.6	21.4	
**Cigarette smoking**				<0.001
Current	33 (84.6)	69.5	94.1	
Previous	4 (10.3)	2.9	24.2	
Never	2 (5.1)	0.6	17.3	
**Severity**				<0.001
Moderate	26 (66.7)	49.8	80.9	
Severe	8 (20.5)	9.3	36.5	
Mild	5 (12.8)	4.3	27.4	
**Initial treatment**				0.002
Itraconazole	29 (74.4)	57.9	87.0	
CMX	10 (25.6)	13.0	42.1	

*Mean ± Standard Deviation. CI, confidence interval; CMX, cotrimoxazole or trimethoprim/sulfamethoxazole; SD, standard deviation. Proportion test: χ^2^ goodness of fit.

†One-sample t test.

### Storage iron

**Fig 2:**
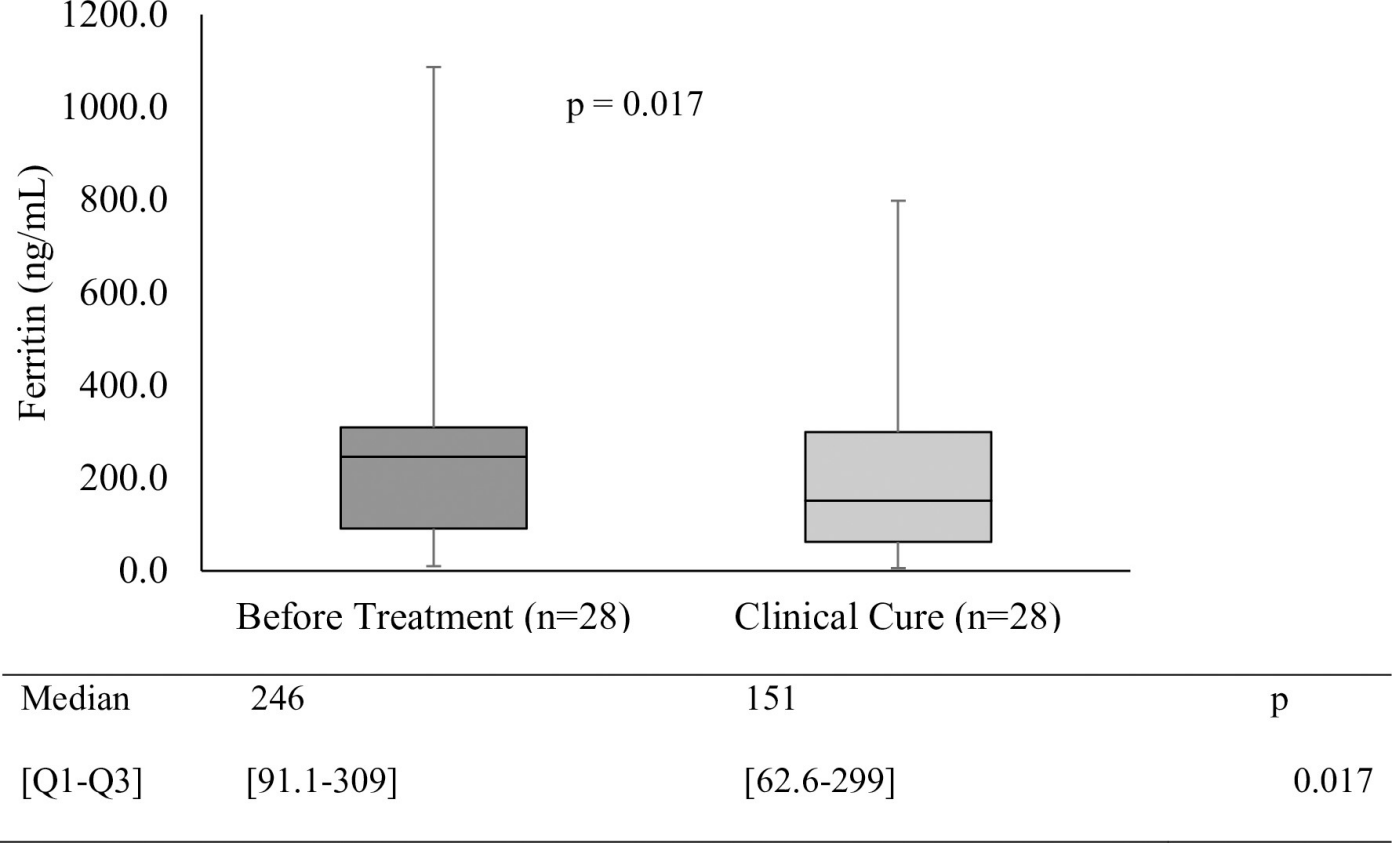
Comparison of median serum ferritin values before treatment (BT) and at the time of clinical cure (CC) of patients with chronic paracoccidioidomycosis. Q1, first quartile; Q3, third quartile. Wilcoxon signed rank test.

Before treatment, the ferritin level of 34 from 39 included patients was evaluated. Nine (26.5%) had high ferritin levels and only two (5.8%) had lower than normal ferritin levels. An analysis of the 28 patients who were evaluated at both time points had decreased ferritin levels. at the time of clinical cure compared to those before treatment (p = 0.017) (**Fig 2**).

### Transport iron

Transport iron was evaluated according to the TSAT level and TIBC. Of the 39 patients evaluated before treatment, 27 (69.2%) had TSAT levels lower than the normal value (<30%) and 8 (20.5%) had TSAT levels <16%. An analysis of the median TSAT values showed no statistical differences between the 30 patients who could be evaluated before treatment and at the time of clinical cure (**Table 3**). Similarly the number of patients with TSAT levels <30% did not decrease (before treatment, n = 19; clinical cure, n = 12; p = 0.071).

**Table 3:** Comparison of the transport iron parameters before treatment and at the time of clinical cure of 30 patients with chronic paracoccidioidomycosis.

Variables	Before Treatment		Clinical Cure		p
	Mean or Median	SD or Q1; Q3	Mean or Median	SD or Q1; Q3	
TSAT (%)	26	18.0;36.8	32.5	24.3; 40.0	0.076
TIBC (μg/dL)	245	100.9	265	85.7	0.398

Q1, first quartile; Q3, third quartile; SD, standard deviation; TIBC, total iron-binding capacity; TSAT, transferrin saturation. The Wilcoxon signed rank test and paired Student’s t test were performed.

The TIBC analysis showed that 22 (56.4%) of 39 individuals had lower than normal values before treatment. At the time of clinical cure, the number of individuals with a TIBC lower than the reference value had not decreased (n = 10 [35.7%]; p = 0.439). An analysis of the mean values before treatment showed that the TIBC was lower than normal; however, it had not increased at the time of clinical cure (**Table 3**).

### Functional iron

The HMG test was performed to evaluate the Hb, RBC, and HCT levels, mean corpuscular volume, and mean corpuscular Hb level to assess functional iron. Additionally, to complement this evaluation, the serum iron and sTfR values and sTfR/log ferritin ratio were calculated.

Anemia, which was considered when the Hb level was <13.0 g/dL for male patients and < 12.0 for female patients, was noted in 46.1% (n = 18) of patients. Among these patients, 61.1% (n = 11) had normocytic and normochromic anemia. The mean Hb level among the 18 patients with anemia was 11.2 g/dL (SD, 1.21 g/dL).

During the healing process, improvement with treatment could be observed by the increase in central values measured at the time of clinical cure compared to those before treatment, except for the sTfR levels and sTfR/log ferritin ratios, which did not differ. (**Table 4**).

**Table 4:** Comparison of functional iron parameter values before treatment and at the time of clinical cure of patients with chronic paracoccidioidomycosis.

Variables	Before Treatment			Clinical Cure			p
	n	Mean or Median	SD or Q1;Q3	n	Mean or Median	SD or Q1;Q3	
Hb	35	13.1	2.2	35	14.5	1.8	<0.001
RBCs	35	4.5	0.6	35	4.8	0.6	0.002
HCT	35	39.9	6.1	35	43.6	5.2	<0.001
MCV	35	89.0	5.7	35	91.0	5.3	0.093
MCH	35	29.3	2.3	35	30.4	2.2	0.008
Iron	32	64.0	37.0;88.5	32	85.5	69.0;109.0	0.004
sTfR	18	21.5	17.4;7.6	18	16.2	6.3;20.2	0.099
sTfR/logFerr	11	8.1	2.8	11	7.6	3.9	0.660

SD, standard deviation; Q1, first quartile; Q3, third quartile; Hb, hemoglobin; RBCs, red blood cells (total erythrocyte count); HCT, hematocrit; MCV, mean corpuscular volume; MHC, mean hemoglobin concentration; sTfR, soluble transferrin receptor; sTfR/log Ferr index, soluble transferrin receptor index/ferritin logarithm. The Wilcoxon signed rank test and paired Student’s t test were performed.

### Inflammatory process

**Fig 3:**
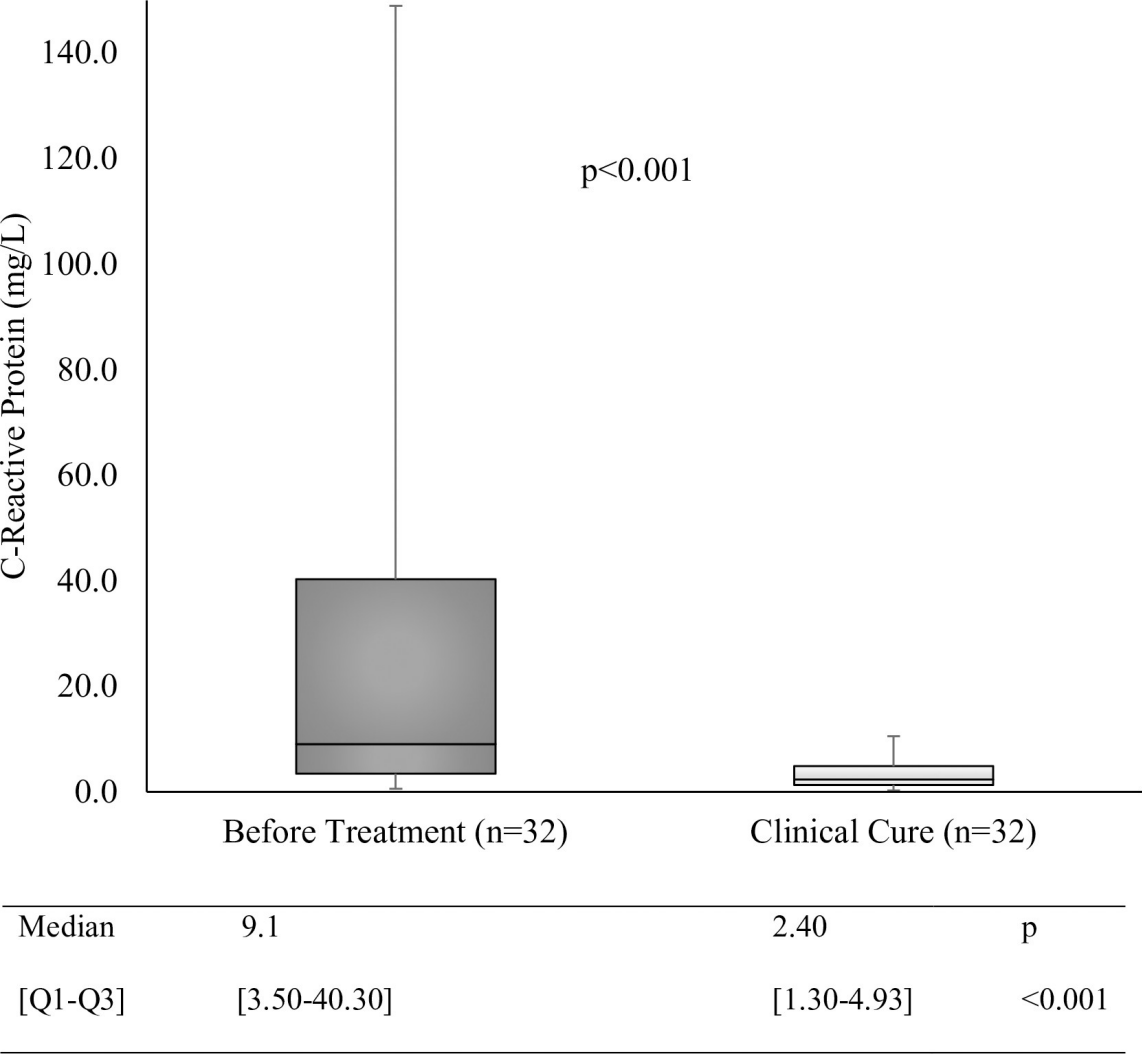
Boxplot of the inflammatory process evaluation involving CRP during different stages of PCM. Q1, first quartile; Q3, third quartile; BT, before treatment; CC, clinical cure. Wilcoxon signed rank test.

Prior to undergoing treatment, 20 of the 32 patients (62.5%) had CRP values that exceeded the normal range. At the point of achieving clinical cure, there was no significant decrease in the percentage of patients with values outside the normal range (n = 7 [21.9%]; p = 0.225). However, an analysis of the median PCR values for each disease stage revealed a significant reduction in values at the time of clinical cure compared to those before treatment (**Fig 3**).

**Fig 4:**
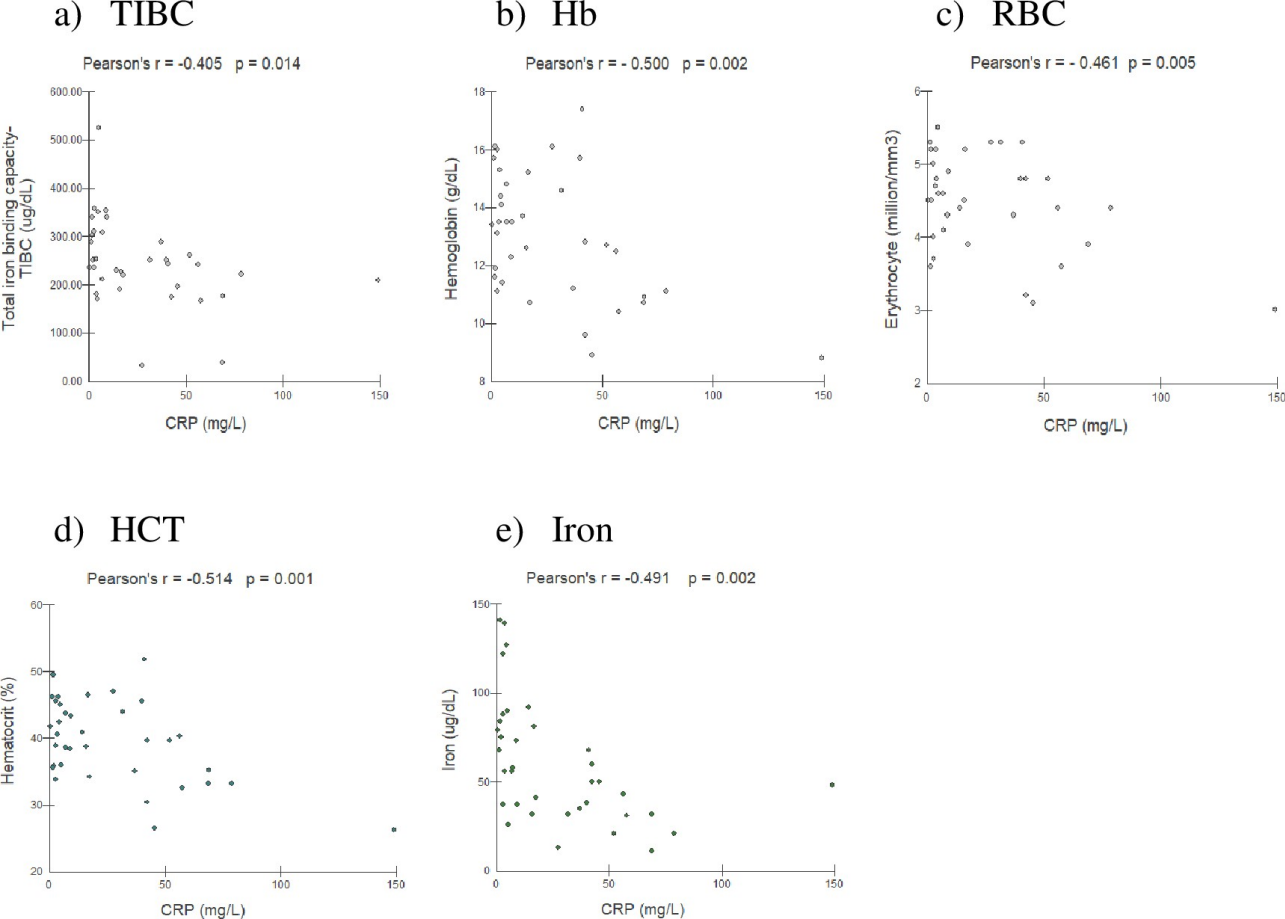
Pearson’s correlations of C-reactive protein (CRP) with variables used to evaluate the iron forms of patients with chronic paracoccidioidomycosis before treatment (n = 36 patients). (a) Correlation of CRP with the total iron-binding capacity (TIBC). (b) Correlation of CRP with hemoglobin (Hb). (c) Correlation of CRP with red blood cells (RBCs; total erythrocyte count). (d) Correlation of CRP with hematocrit (HCT).

Weak to moderate indirect correlations between CRP levels during active disease (before treatment) and the TIBC (r = -0.405; p = 0.014), Hb level (r = -0.500; p = 0.002), RBC level (r = -0.461; p = 0.005), HCT level (r = -0.514; p = 0.001), and iron level (r = -0.491; p = 0.002) were observed (**Fig 4**). At the time of clinical cure, no correlations were observed between the CRP levels and these variables.

An analysis of the stored iron and functional iron parameters, as well CRP levels, before treatment showed differences between severe and mild or moderate disease cases (**Table 5**).

**Table 5:** Analysis of iron parameters and CRP levels according to disease severity of patients with chronic paracoccidioidomycosis before treatment.

Variables	Severe (n = 08)	Mild or Moderate (n = 31)	p
	Mean (SD) or	Mean (SD) or	
	Median [Q1;Q3]	Median [Q1;Q3]	
**Storage iron**			
Ferritin	448 [308;514]	199 [90.4;290]	0.007
**Transport iron**			
TSAT	18 [14.3;22.8]	27 [18;38.2]	0.051
TIBC	215 [191;245]	241 [201;310]	0.258
**Functional iron**			
RBCs	4.05 (0.81)	4.60 (0.57)	0.031
Hb	11.8 (3.1)	13.5 (1.8)	0.050
HCT	35.8 (8.8)	41.0 (4.8)	0.029
Iron	40.4 (14.5)	68.1 (35.5)	0.039
**CRP**	51.7 [40.8;71.4]	7.06 [2.90;20.1]	<0.001

Q1, first quartile; Q3, third quartile; TSAT, transferrin saturation; TIBC, total iron-binding capacity; RBC, red blood cells (total erythrocyte count); Hb, hemoglobin; HCT, hematocrit; CRP, C-reactive protein. The Mann-Whitney U test and independent Student’s t test were performed.

This study included 33 smokers, 4 ex-smokers and 2 non-smoke. A comparison of the medians of the variables studied revealed that there was no difference between these subgroups.

## Discussion

The acquisition of iron by microorganisms is an important virulence mechanism [17], and it has been reported with *Paracoccidioides* spp. [9, 18]. Additionally, human organisms actively control intracellular and systemic iron levels in a way that can contain infection and/or microbial persistence. Therefore, this study compared iron metabolism parameters before treatment and at the time of clinical cure, analyzed them based on PCM severity, and correlated them with the intensity of the inflammatory process of patients with chronic PCM.

The demographic and clinical characteristics of the patients corresponded to those reported by other case series studies of chronic PCM that involved mostly male patients who were older than age 40 years, smokers, rural workers, and those who had moderate PCM [19–23]. Therefore, this sample is representative of the population of PCM patients and allows the generalization of our results.

More than one quarter of patients had high serum ferritin levels before treatment. Moreover, the median ferritin values decreased at the time of clinical cure, indicating that PCM may have interfered with this parameter. Although ferritin exists in large amounts in the liver and spleen, only a small amount is detected in the circulation. However, serum quantification is a common and non-invasive method that provides an accurate measurement of storage iron [24]. High serum ferritin levels may be related to not only increased storage iron but also inflammatory activity. Because serum ferritin is an acute-phase protein in inflammatory and infectious processes and in cancer, the production of apoferritin is increased because of the stimulation of interleukins (ILs), such as IL-1 and IL-6 [25]. Increases in these ILs have been observed in patients with PCM [26].

To assess the transport iron, we evaluated the TIBC and TSAT level. The TIBC is an indirect measure of circulating transferrin, which is reduced during inflammatory processes, whereas the TSAT level can be low or normal [11]. Before treatment, the patients in this study had median TIBC and TSAT values that were below the lower limit of normal, indicating a transport iron deficit. However, antifungal therapy did not affect these parameters. A weak negative correlation was observed between the TIBC and CRP level, but no correlation was observed between the TSAT and CRP levels. Additionally, no difference in the TIBC and TSAT levels in severe and mild or moderate cases was observed. In the context of chronic PCM, it appears that the parameters related to transport iron (TIBC and TSAT) are less affected than those associated with storage iron and functional iron. This may be due to the lower intensity of the inflammatory process in the chronic form of the disease, which may not be severe enough to impede transferrin production.

A TSAT level <16% is considered indicative of iron deficiency in erythropoiesis; however, the use of this value for this parameter has limitations because both iron levels and the TIBC are lower with inflammatory processes [12, 24]. During our study, TSAT values <16% were found in 8 (20.5%) patients before treatment. However, only one of them had confirmed iron deficiency anemia, which is characterized by low Hb, ferritin, and TSAT levels [27]. A study of the effect of PCM treatment on iron metabolism showed that specific treatment quickly leads to a return to normal values or improvement in iron metabolism, as described for the TIBC, which was lower and normalized soon after antifungal treatment was started [28].

In clinical practice, the HMG test results and serum iron level are the first indicators of a change in the iron status. Although our study showed that the parameter values were slightly or not altered before treatment, it was possible to infer that PCM interferes with functional iron because these values improved after treatment and were associated with PCM severity. Additionally, a moderate correlation was observed between Hb, RBC, HCT, iron, and CRP levels. Furthermore, similar to our results, tuberculosis patients with active disease had lower mean values of these variables [29].

Approximately 65% of the total body iron in circulation in humans is linked to Hb [7], and reduced Hb with chronic infectious diseases is attributable to the limitation of erythropoiesis caused by regulators such as IL-1 and tumor necrosis factor-α [30]. Hb is a source of iron for *Paracoccidioides* spp., which removes iron by hemolytic activity and internalizes protoporphyrin rings through receptor-mediated pathways [31]. Other fungi are also capable of removing iron from Hb, such as *Candida albicans*, through a similar mechanism [32].

During infection, competition between the pathogen and host for iron uptake involves strategic mechanisms. Therefore, in response to infection, human organisms activate the immune system, thus inducing changes in circulating iron and leading to iron storage in macrophages and decreased absorption of iron from the diet by enterocytes [33]. Furthermore, several inflammatory cytokines are released by immune system cells, and they contribute to alterations in iron metabolism [34]. Despite the host-generated deprivation of iron, all fungi, including *Paracoccidioides* spp., have specific mechanisms for iron uptake through the production of molecules with high affinity for iron, siderophores, iron uptake pathways through reductive iron assimilation, or even Hb receptors [9, 35, 36]. Therefore, an inflammatory response associated with infection alters the availability of circulating iron, thus increasing its intracellular storage and leading to hypoferremia and iron-restricted erythropoiesis, as observed during our study, because these changes can contribute to anemia of inflammation [37].

The sTfR level is a good indicator of the iron status in the absence of systemic influences; therefore, we analyzed sTfR levels before treatment and at the time of clinical cure. Our results showed that the median sTfR values before treatment were normal and did not decrease after treatment, suggesting that the body iron is normal; however, it is distributed from functional iron to storage iron. A high sTfR level can indicate iron deficiency [38, 39]. During the storage iron reduction phase, the sTfR level does not change. However, when there is a decrease in functional iron, the synthesis of the transferrin receptor is stimulated; consequently, the sTfR level increases. This parameter is mainly recommended for differentiating iron deficiency anemia from inflammation anemia/chronic disease anemia because the value is increased with iron deficiency anemia but normal with inflammation anemia [40–42]. Studies of pulmonary tuberculosis patients suggested that high sTfR levels in patients with anemia indicate probable iron deficiency and recommended that the sTfR concentration of patients with anemia and an infectious/inflammatory process should be examined [29].

Another good indicator of iron deficiency with differential anemia is the sTfR/log ferritin ratio [43]. During our study, the sTfR/log ferritin ratio as well as sTfR level were normal before treatment and did not significantly change after treatment, reinforcing the belief that anemia with chronic PCM is inflammation anemia.

This study had some limitations. The number of patients was low. However, our institution encounters approximately 15 new cases every year and most of the patients do not undergo follow-up after clinical improvement; therefore, it is difficult to pair of some variables–before treatment and after clinical cure. Furthermore, to assess the frequency of abnormalities before treatment we considered reference values of healthy adults. It is important to note that our study population consisted of predominantly smokers, and it is well known that hemoglobin, erythrocytes, hematocrit, and ferritin levels are generally higher in smokers and individuals with chronic obstructive pulmonary disease [44]. This means that the observed abnormalities before treatment would have been even more pronounced were it not for the interference of smoking. However, because the majority of patients were smokers, and because no differences in the variables of smokers, ex-smokers, and non-smokers were observed, smoking must not have compromised the analyses before treatment and clinical cure.

In conclusion, PCM interferes with iron metabolism by shifting functional iron to storage iron, as revealed by the increased functional iron parameters and decreased serum ferritin level after clinical cure. Moreover, these parameters were more altered in severe cases than in mild or moderate cases. Although anemia caused by iron deficiency can be present in the chronic form of pulmonary comorbidity (PCM), as evident in one of our cases, it is important to note that anemia of inflammation is more prevalent. Hence, a thorough evaluation of iron parameters is crucial to prevent inappropriate supplementation of this mineral and promote better clinical management of PCM.

The limited current understanding of the impact of PCM on systemic iron metabolism underscores the need for further investigation. Our study raises important questions that warrant additional research to elucidate the potential role of altered iron metabolism in PCM pathogenesis, as well as the possible involvement of hepcidin, which is a key regulator of iron homeostasis, in chronic PCM-related anemia. A deeper understanding of the intricate interplay between PCM and iron metabolism could yield novel insights and therapeutic strategies for managing PCM.

## Supporting information

S1 Checklist STROBE statement—Checklist of items that should be included in reports of *cross-sectional studies*. (DOCX)

S1 Dataset Dataset containing variables of the study. (XLS)
